# Correction: Comparison of risk models for mortality and cardiovascular events between machine learning and conventional logistic regression analysis

**DOI:** 10.1371/journal.pone.0223931

**Published:** 2019-10-10

**Authors:** The PLOS ONE Staff

## Notice of republication

An incomplete, earlier version of this article was published in error. The publisher apologizes for the error. This article was republished on October 1, 2019 to correct for this error. Please download the article again to view the correct version. The originally published, uncorrected article and the republished, corrected article are provided here for reference.

## Supporting information

S1 File Originally published, uncorrected article. (PDF)

S2 File Republished corrected article. (PDF)
