# Supplementary material for: Correction: Comparison of risk models for mortality and cardiovascular events between machine learning and conventional logistic regression analysis
Source: PLoS One. 2019 Oct 10;14(10):e0223931. doi: 10.1371/journal.pone.0223931 (PMC6786577; doi:10.1371/journal.pone.0223931)
Supplement: S2 File — (PDF) [file pone.0223931.s002.pdf]

PLOS ONE

RESEARCH ARTICLE

Comparison of risk models for mortality and cardiovascular events between machine learning and conventional logistic regression analysis

Shinya Suzuki[1]*, Takeshi Yamashita[1], Tsuyoshi Sakama[2], Takuto Arita[1], Naoharu Yagi[1], Takayuki Otsuka[1], Hiroaki Semba[1], Hiroto Kano[1], Shunsuke Matsuno[1], Yuko Kato[1], Tokuhisa Uejima[1], Yuji Oikawa[1], Minoru Matsuhama[3], Junji Yajima[1]

1 Department of Cardiovascular Medicine, The Cardiovascular Institute, Tokyo, Japan, **2** Sigmaxyz, Inc, Tokyo, Japan, **3** Department of Cardiovascular Surgery, The Cardiovascular Institute, Tokyo, Japan

* sinsuz-tky@umin.net

Check for updates

OPEN ACCESS

Citation: Suzuki S, Yamashita T, Sakama T, Arita T, Yagi N, Otsuka T, et al. (2019) Comparison of risk models for mortality and cardiovascular events between machine learning and conventional logistic regression analysis. PLoS ONE 14(9): e0221911. https://doi.org/10.1371/journal.pone.0221911

Editor: Yu Ru Kou, National Yang-Ming University, TAIWAN

Received: June 11, 2019

Accepted: August 16, 2019

Published: September 9, 2019

Copyright: © 2019 Suzuki et al. This is an open access article distributed under the terms of the Creative Commons Attribution License, which permits unrestricted use, distribution, and reproduction in any medium, provided the original author and source are credited.

Data Availability Statement: Data cannot be shared publicly because of a lack of such description in the study protocol and informed consent. Data are available from the Ethics Review Committee at the Cardiovascular Institute for researchers who meet the criteria for access to confidential data. (contact via Kazumi Matsuda, E-mail: matsuda@cvi.or.jp).

Funding: This work was supported by AMED (17ek0210082): the Practical Research Project for

Abstract

Aims

Non-linear models by machine learning may identify different risk factors with different weighting in comparison to conventional linear models.

Methods and results

The analyses were performed in 15,933 patients included in the Shinken Database (SD) 2004–2014 ($n = 22{,}022$) for whom baseline data of blood sampling and ultrasound cardiogram and follow-up data at 2 years were available. Using non-linear models with machine learning software, 118 risk factors and their weighting of risk for all-cause mortality, heart failure (HF), acute coronary syndrome (ACS), ischemic stroke (IS), and intracranial hemorrhage (ICH) were identified, where the top two risk factors were albumin/hemoglobin, left ventricular ejection fraction/history of HF, history of ACS/anti-platelet use, history of IS/deceleration time, and history of ICH/warfarin use. The areas under the curve of the developed models for each event were 0.900, 0.912, 0.879, 0.758, and 0.753, respectively.

Conclusion

Here, we described our experience with the development of models for predicting cardiovascular prognosis by machine learning. Machine learning could identify risk predicting models with good predictive capability and good discrimination of the risk impact.

Introduction

Risk prediction models for cardiovascular disease (CVD) are generally based on an assumption that each risk factor is linearly associated with CVD outcomes.[1, 2] Such models may

Life-Style related Diseases including Cardiovascular Diseases and Diabetes Mellitus from Japan Agency for Medical Research and Development, AMED (https://www.amed.go.jp/en/index.html). AMED did not have any roles in the study design, data collection and analysis, decision to publish, or preparation of the manuscript.

Competing interests: Dr. Suzuki received research funding from Mitsubishi Tanabe Pharm, and Daiichi Sankyo. Dr. Yamashita has received research funds and/or lecture fees from Daiichi Sankyo, Bayer Yakuhin, Bristol-Myers Squibb, Pfizer, Nippon Boehringer Ingelheim, Eisai, Mitsubishi Tanabe Pharm, Ono Pharmaceutical, and Toa Eiyo. These do not alter our adherence to PLOS ONE policies on sharing data and materials.

oversimplify complex relationships, which potentially include both non-linear associations and non-linear interactions. Therefore, better approaches to develop risk models which reflect the real relationship between risk factors and outcomes are necessary.

Machine learning (ML) offers an alternative approach for development of prediction models. ML is a scientific research standing at the intersection of statistics and computer science, which depends on efficient computing algorithms. The importance of ML has been recognized through the challenges of building statistical models from massive data sets which required computational methods. [3] ML may detect the complex and non-linear interactions between variables by minimizing the error between predicted and observed outcomes.[4]

To date, although there have been several investigations comparing model development for prognostic assessment of CVD between ML and commonly used statistical methods, no large-scale investigations have been reported in a Japanese cohort. This study was performed to examine whether there are any differences between modelling by ML and by logistic regression analysis using a single-centre cohort in a cardiovascular hospital in Japan.[5, 6]

Methods

Study population

The Shinken Database includes all patients newly visiting the Cardiovascular Institute in Tokyo, Japan ('Shinken' is a Japanese abbreviation for the name of the hospital), excluding foreign travelers and patients with active cancer. This hospital-based database was established to investigate the prevalence and prognosis of CVD.[5, 6] Our hospital is a cardiology specialized hospital in an urban area of Japan, Tokyo. The patients seen were not only local residents but also referred from other clinics for treatment of CVD. The attending physicians were all cardiologists or cardiothoracic surgeons.

The registry began in June 2004, and patients have been continually registered in the database annually. A total of 29,832 patients were registered between June 2004 and March 2016. Of these, 15,993 patients whose 2-year follow-up data were available were analysed in the present study.

Ethics

The ethics committee of the Cardiovascular Institute approved this study, and all patients provided written informed consent.

Data collection

After obtaining an electrocardiogram and chest X-ray, the cardiovascular status of the patients was evaluated by echocardiography, exercise test, 24-hour Holter recordings, and blood laboratory data from the initial visit. In addition to gender, age, height, weight, and medications prescribed at the initial visit, we collected data on CVD, including heart failure (HF; New York Heart Association class $\geq$ 2), valvular heart disease (moderate or severe stenosis or regurgitation using echocardiography), coronary artery disease (diagnosed by angiography or scintigraphy), hypertrophic and dilated cardiomyopathy (diagnosed by echocardiography or magnetic resonance imaging), left ventricular non-compaction (diagnosed by echocardiography), and history of a disabling cerebral infarction or transient ischemic attack (diagnosed by computed tomography or magnetic resonance imaging). The presence of cardiovascular risk factors, including hypertension (use of anti-hypertensive agents, systolic blood pressure $\geq$ 140 mmHg, or diastolic blood pressure $\geq$ 90 mmHg on admission), diabetes mellitus (use of oral hypoglycemic agents or insulin, or glycosylated hemoglobin $\geq$ 6.5%), dyslipidemia (use of a statins or

drugs for lowering triglycerides, low-density lipoprotein cholesterol $\geq$ 140 mg/dL, high-density lipoprotein cholesterol < 40 mg/dL, or triglycerides $\geq$ 150 mg/dL), chronic kidney disease (estimated glomerular filtration rate < 60 mL/minute/m^2), chronic obstructive pulmonary disease, and use of anti-coagulant and anti-platelet medications were determined. Body mass index (BMI) was calculated as weight in kilograms divided by height in meters squared. The glomerular filtration rate (GFR) was estimated using the new Japanese coefficient for the modified isotope dilution mass spectrometry (IDMS)-traceable 4-variable Modification of Diet in Renal Disease (MDRD) study equation (GFR = 194 × serum creatinine (SCr) − 1.094 × Age − 0.287 × 0.739 [if female]).[7]

Parameters assessed for the prediction models

Of the parameters obtained for the database, 118 parameters were used for development of the prediction models (Table 1). Patient parameters (including age, sex, smoking habit, and drinking habit), diagnosis of comorbidities, and information regarding medications were obtained. Blood pressure, laboratory data, and parameters of ultrasound cardiogram were measured in almost all of the patients; however, in some patients, measurements were not performed incidentally (i.e., at the patient's requires or at the discretion of the attending physician), and some of the parameters were lacking for technical reasons. As described below, in the ML method, missing values were complemented by a automatically selected prespecified algorithm, while data with missing values were excluded from logistic regression analysis with commonly used statistical software.

Patient outcome

The patient outcomes in the present study included the following six events: all-cause mortality, cardiovascular events, HF events, acute coronary syndrome (ACS) events, ischemic stroke

Table 1. Parameters assessed in the prediction models.

Category	Number of parameters	Parameters
Patient information	9	age, sex, body height, body weight, body mass index, systolic blood pressure, diastolic blood pressure, smoking habit, drinking habit
Comorbidity	43	hypertension, dyslipidemia, diabetes mellitus, uric acid, chronic kidney disease, anemia, heart failure, stable angina pectoris, vasospastic angina pectoris, acute coronary syndrome, old myocardial infarction, silent myocardial ischemia, ischemic cardiomyopathy, atherosclerosis obliterans, history of percutaneous coronary intervention, history of coronary artery bypass graft, mitral stenosis, mitral regurgitation, aortic stenosis, aortic regurgitation, tricuspid regurgitation, history of heart valve replacement, dilated cardiomyopathy, hypertrophic cardiomyopathy, dilated-phase hypertrophic cardiomyopathy, hypertensive heart disease, congenital heart disease, aortic dissection, aortic aneurism, sick sinus syndrome, atrioventricular block (II or more degrees), atrial fibrillation, atrial tachycardia/atrial flutter, ventricular fibrillation/sustained ventricular tachycardia, non-sustained ventricular tachycardia, history of catheter ablation, permanent pacemaker/implanted cardioverter defibrillator/cardiac resynchronization therapy implantation, history of symptomatic ischemic stroke or transient ischemic attack, history of intracranial hemorrhage, hyperthyroidism, chronic obstructive pulmonary disease, chronic hemodialysis
UCG parameters	14	interventricular septum thickness, posterior wall thickness, left ventricular end-diastolic diameter, left ventricular diameter at end systole, left ventricular ejection fraction, left atrial dimension, mitral regurgitation, aortic regurgitation, tricuspid regurgitation, right ventricular systolic pressure, E, A, E/A, deceleration time
Laboratory data	26	total protein, albumin, blood urea nitrogen, creatinine, estimated glomerular filtration rate, uric acid, sodium, potassium, chlorine, triglyceride, total cholesterol, aspartate aminotransferase, alanine transaminase, lactate dehydrogenase, creatine kinase, blood sugar, brain natriuretic peptide, white blood cell count, red blood cell count, hemoglobin, hematocrit, red cell distribution width, platelet count, mean platelet volume, plateletcrit, platelet distribution width
Medications	26	hypertensive drugs, beta blockers, calcium blockers, angiotensin converting enzyme inhibitors, angiotensin-II receptor blockers, alfa blockers, sodium glucose transporter-2 inhibitors, insulin, statin, eicosapentenoic acid drugs, diuretics, class I anti-arrhythmic drugs, carvedilol, bisoprolol, atenolol, class III anti-arrhythmic drugs, class IV anti-arrhythmic drugs, digitalis, antiplatelet, warfarin, direct oral anticoagulants, anti-thyroid drugs, thyroid drugs, non-steroidal anti-inflammatory drugs, benzodiazepines, non-benzodiazepine

UCG: ultrasound cardiogram

https://doi.org/10.1371/journal.pone.0221911.t001

(IS) events, and intracranial hemorrhage events. Cardiovascular events were defined as a composite of four events (HF events, ACS events, IS events, and intracranial hemorrhage events). Each event comprising a cardiovascular event was determined when it required hospitalization. We used the follow-up data with a maximum observation period of 2 years.

Data analysis by machine learning

We developed linear and non-linear models using an automated ML platform, DataRobot.[8] More than 3,000 procedure sets of data processing, feature engineering, and ML algorithm, including Support Vector Machine, Elastic Net Classifier, Regularized Logistic Regression, Stochastic Gradient Descent Classifier, Neural Network Classifier, etc., are developed from its repository. The software automatically chooses and executes suitable procedure sets when investigating the patterns in data. All of the developed models were verified by cross-validation and sorted by the selected evaluation metric, e.g., the area under the curve (AUC).

1) Data preprocessing. From a large number of data preprocessing approaches, the following approaches were automatically selected in the final models: imputing missing values, one-hot encoding for categorical values, standardization for numerical values, and creating new parameters by unsupervised learning of original parameters. Missing numerical values were imputed based on the medians of values in its parameters, and missing categorical values were treated as their own categorical level and given their own parameters. Categorical values were converted to many binary parameters by one-hot encoding if needed. For some models, numerical values were standardized in each parameter by subtracting the mean and dividing by the standard deviation. Moreover, some new parameters were created internally by summarizing original parameters with an unsupervised learning method.

2) Model validation. All developed models were validated by cross-validation and holdout, using the AUC of the receiver-operating characteristic (ROC) curve as the evaluation metric. Before developing models, 20% of the dataset was randomly selected as the holdout, which was never used in training or validation. The remaining data were randomly divided into five mutually exclusive folds of data, four of which were used together for training, with the final fold used for validation.[9] Models were trained five times per algorithm, with each fold used once for validation. Cross-validation scores were calculated by taking the mean of AUC of the five possible validation folds.[10] Random selection was performed in cross-validation and holdout by stratified sampling, which holds the ratio of positive and negative cases. Finally, models were validated on the holdout to demonstrate the generalization performance to new data. As the holdout was taken as a single sample, no confidence intervals were calculated.

3) Permutation Importance. The relative importance of a parameter in the models was assessed using the permutation importance (PI), as described by Breiman.[11] This method is widely used in ML as it can be applied to both linear and non-linear models. To calculate the PI of a parameter in a model, its values in the validation data were randomly shuffled (reordered), keeping other parameters the same as before. If it has considerable importance on the outcome, the resulting performance score in the evaluation metric should decline significantly. We calculated the PI of all parameters and divided by the maximum ratio of the resulting performance scores on the original scores to normalize and compare among different models. The calculation was conducted several times to ensure stability in random shuffling.

4) Partial dependence. To understand how the changes in values of a parameter affect the outcome, we constructed partial dependence plots as described by Friedman.[12] To construct the partial dependence plot of a parameter in a model, we calculated predictions from the model after having replaced all the values for the parameter with a constant value and

computing the mean of those predictions. We repeated calculations for many values to observe how the model reacts to changes in the parameter of interest.

Logistic regression analysis by commonly used software

1) Model development. For comparison with prediction modelling by ML, logistic regression analysis was performed with commonly used statistical software (SPSS ver. 19; IBM Corp., Armonk, NY, USA). We used 118 similar parameters, and consecutive variables were assumed to have a linear association with the patient outcomes. The multivariate model was developed with the forward stepwise method. The interactions between parameters were not considered. Data with missing values were excluded from the analysis.

2) Impact of risk factors. Impact of risk factors (IRF) was calculated for each parameter determined in the multivariate models by logistic regression analysis using the following equation: IRF = (Wald statistic for each parameter)/(maximum Wald statistic among parameters in the multivariate model). IRF in the logistic regression model corresponded to the permutation importance in the ML model.

Other statistical methods

Categorical and continuous data are presented as numbers (%) and means ± standard deviation, respectively. Statistical analyses other than ML were performed using SPSS ver. 19 (IBM Corp.). In all analyses, two-sided $P < 0.05$ was taken to indicate statistical significance.

Results

Patient characteristics

The patient characteristics of the study population (n = 15,933) are shown in Table 2. The mean age was 61 ± 14 years, and the population included 10,352 males (65%). The rates of hypertension, dyslipidemia, and diabetes were 51%, 39%, and 20%, respectively, whereas those of HF, ischemic heart disease, valvular heart disease, cardiomyopathy, and atrial fibrillation were 18%, 26%, 14%, 9%, and 18%, respectively.

Incidence rates of patient outcomes

The incidence rates of patient outcomes (percentage for number of study population) are shown in Table 3. All-cause mortality occurred in 217 patients (1% within 2 years),

Table 2. Patient characteristics.

**Total, *n* = 15,933**	
Age, years	61 ± 14
Male	10,352 (65)
Hypertension	8,110 (51)
Dyslipidemia	6,250 (39)
Diabetes	3,154 (20)
Heart failure	2,831 (18)
Ischemic heart disease	4,133 (26)
Valvular heart disease	2,197 (14)
Cardiomyopathy	1,352 (9)
Atrial fibrillation	2,805 (18)

Data are presented as *n* (%) of patients or mean ± standard deviation.

https://doi.org/10.1371/journal.pone.0221911.t002

cardiovascular events in 786 (5%), HF events in 417 (3%), ACS events in 247 (2%), IS events in 95 (0.6%), and intracranial hemorrhage events in 59 (0.4%).

Comparison of prediction models

1) All-cause mortality. Among the ML models, a model with Nystroem Kernel SVM Classifier had the largest AUC for all-cause mortality (0.900). In this model, the top five parameters determined by PI were albumin (100, reference), hemoglobin (78), aortic aneurism (44), BMI (43), and maintenance hemodialysis (43). Albumin, hemoglobin, and BMI showed linear relationships with all-cause mortality (Table 4A, Figs 1A and 2A).

In the logistic regression model, AUC for all-cause mortality was 0.881. In this model, the top five parameters determined by IRF were albumin (100, reference), age (36), total protein (29), dyslipidemia (28), and carvedilol use (25) (Fig 1A).

2) Cardiovascular events. Among the ML models, a model with Random Forest Classifier (Tree-based Algorithm) had the largest AUC for cardiovascular events (0.848). In this model, the top five parameters determined by PI were HF (100, reference), history of ACS (76), left ventricular ejection fraction (72), estimated glomerular filtration rate (57), and mitral regurgitation (degree determined by ultrasound cardiogram) (42). Mitral regurgitation showed a linear relationship with cardiovascular events, whereas left ventricular ejection fraction and estimated glomerular filtration rate showed non-linear relationships (Table 4B, Figs 1B and 2B).

In the logistic regression model, the AUC for cardiovascular events was 0.831. In this model, the top five parameters determined by IRF were history of ACS (100, reference), HF (57), left ventricular ejection fraction (30), tricuspid regurgitation (degree determined by ultrasound cardiogram) (17), and statin use (17) (Fig 1B).

3) Heart failure events. Among the ML models, a model with Random Forest Classifier (Tree-based Algorithm) had the largest AUC for HF event (0.912). In this model, the top five parameters determined by PI were left ventricular ejection fraction (100, reference), HF (93), age (57), left ventricular dimension at end-diastole (57), and left atrial dimension (49). Left ventricular ejection fraction, age, left ventricular dimension at end-diastole, and left atrial dimension showed non-linear relationships (with a threshold) with HF events (Table 4C, Figs 1C and 2C).

In the logistic regression model, the AUC for HF events was 0.907. In this model, the top five parameters determined by IRF were left ventricular dimension at end-systole (100, reference), diuretic use (77), HF (71), direct oral anti-coagulant use (61), and left atrial dimension (58) (Fig 1C).

4) Acute coronary syndrome events. Among the ML models, a model with Elastic-Net Classifier had the largest AUC for ACS events (0.879). In this model, the top five parameters

Table 3. Incidence rates of patient outcomes.

Total, *n* = 15,933	Incidence rate within 2 years
All-cause mortality	217 (1)
Cardiovascular events	786 (5)
Heart failure events	417 (3)
Acute coronary syndrome	247 (2)
Ischemic stroke events	95 (0.6)
Intracranial hemorrhage	59 (0.4)

Data are presented as *n* (%).

https://doi.org/10.1371/journal.pone.0221911.t003

Table 4. Top five parameters for patient outcome.

	Machine learning		Logistic regression model	
A. All-cause death				
	Model	AUC	Model	AUC
	Support vector machine	0.900	——	0.881
	Parameters	IRF (%)	Parameters	PI (%)
1	Albumin	100	Albumin	100
2	Hemoglobin	78	Age	36
3	Aortic aneurism	44	Total protein	29
4	Body mass index	43	Dyslipidemia	28
5	Hemodialysis	43	Carvedilol use	25
B. Cardiovascular events				
	Model	AUC	Model	AUC
	Random forest	0.848	——	0.831
	Parameters	IRF (%)	Parameters	PI (%)
1	Heart failure	100	History of acute coronary syndrome	100
2	History of acute coronary syndrome	76	Heart failure	57
3	Left ventricular ejection fraction	72	Left ventricular ejection fraction	30
4	Estimated glomerular filtration rate	57	Tricuspid regurgitation (degree)	17
5	Mitral regurgitation (degree)	42	Statin use	17
C. Heart failure events				
	Model	AUC	Model	AUC
	Random forest	0.912	——	0.907
	Parameters	IRF (%)	Parameters	PI (%)
1	Left ventricular ejection fraction	100	Left ventricular dimension at end-systole	100
2	Heart failure	93	Diuretics use	77
3	Age	57	Heart failure	71
4	Left ventricular dimension at end-diastole	57	Direct oral anticoagulant	61
5	Left atrial dimension	49	Left atrial dimension	58
D. Acute coronary syndrome events				
	Model	AUC	Model	AUC
	Elastic-Net	0.879	——	0.884
	Parameters	IRF (%)	Parameters	PI (%)
1	History of acute coronary syndrome	100	History of acute coronary syndrome	100
2	Antiplatelet use	26	Antiplatelet use	23
3	Diuretics use	18	Stable angina	20
4	Heart failure	17	Old myocardial infarction	9
5	Angiotensin receptor-II blocker	10	Creatinine	8
E. Ischemic stroke events				
	Model	AUC	Model	AUC
	Support vector machine	0.758	——	0.757
	Parameters	IRF (%)	Parameters	PI (%)
1	History of ischemic stroke or TIA	100	History of ischemic stroke or TIA	100
2	Deceleration time	98	Systolic blood pressure	52
3	History of intracranial hemorrhage	76	Blood glucose	51
4	Diastolic blood pressure	48	Aortic aneurism	30
5	Left ventricular ejection fraction	34	Tricuspid regurgitation	28
F. Intracranial hemorrhage events				
	Model	AUC	Model	AUC

(*Continued*)

Table 4. (Continued)

	Machine learning		Logistic regression model	
	Elastic-Net	0.753	——	0.726
	Parameters	IRF (%)	Parameters	PI (%)
1	History of intracranial hemorrhage	100	History of intracranial hemorrhage	100
2	Warfarin use	65	Tricuspid regurgitation (degree)	79
3	Interventricular septal thickness	37	Interventricular septal thickness	50
4	Dilated-phase hypertrophic cardiomyopathy	29	Estimated glomerular filtration rate	33
5	Sick sinus syndrome	29	History of coronary artery bypass graft	31

Abbreviations: AUC; area under the curve, IRF; impact of risk factors, PI; permutation importance, TIA; transient ischemic attack.

https://doi.org/10.1371/journal.pone.0221911.t004

determined by PI were history of ACS (100, reference), anti-platelet use (26), diuretic use (18), HF (17), and angiotensin-receptor-II blocker use (10) (Table 4D, Figs 1D and 2D).

In the logistic regression model, the AUC for ACS events was 0.884. In this model, the top five parameters determined by IRF were history of ACS (100, reference), anti-platelet use (23), stable angina (20), old myocardial infarction (9), and creatinine (8) (Fig 1D).

5) Ischemic stroke events. Among the ML models, a model with Nystroem Kernel SVM Classifier (Regularized Linear Model) had the largest AUC for IS events (0.758). In this model, the top five parameters determined by PI were history of IS or transient ischemic attack (100, reference), deceleration time (98), history of intracranial hemorrhage (76), diastolic blood pressure (48), and left ventricular ejection fraction (34). Deceleration time, diastolic blood pressure, and left ventricular ejection fraction showed linear relationships with IS events (Table 4E, Figs 1E and 2E).

In the logistic regression model, the AUC for IS events was 0.757. In this model, the top five parameters determined by IRF were history of IS or transient ischemic attack (100, reference), systolic blood pressure (52), blood glucose (51), aortic aneurism (30), and tricuspid regurgitation (28) (Fig 1E).

6) Intracranial hemorrhage events. Among the ML models, a model with Elastic-Net Classifier had the largest AUC for intracranial hemorrhage events (0.753). In this model, the top five parameters determined by PI were history of intracranial hemorrhage (100, reference), warfarin use (65), interventricular septal thickness (37), dilated phase hypertrophy cardiomyopathy (29), and sick sinus syndrome (29). Interventricular septal thickness showed a linear relationship with intracranial hemorrhage events (Table 4F, Figs 1F and 2F).

In the logistic regression model, the AUC for intracranial hemorrhage events was 0.726. In this model, the top five parameters determined by IRF were history of intracranial hemorrhage (100, reference), tricuspid regurgitation (degree determined by echocardiogram) (79), interventricular septal thickness (50), estimated glomerular filtration rate (33), and history of coronary artery bypass graft (31) (Fig 1F).

Discussion

Major findings

In this analysis, we developed prediction models for six prognostic outcomes related to CVD by ML (ML model) using ensemble modelling software (DataRobot) and logistic regression analysis (LR model) with commonly used statistical software (SPSS). The AUCs for each prognostic outcome were mostly similar between ML and LR models. Interestingly, the parameter

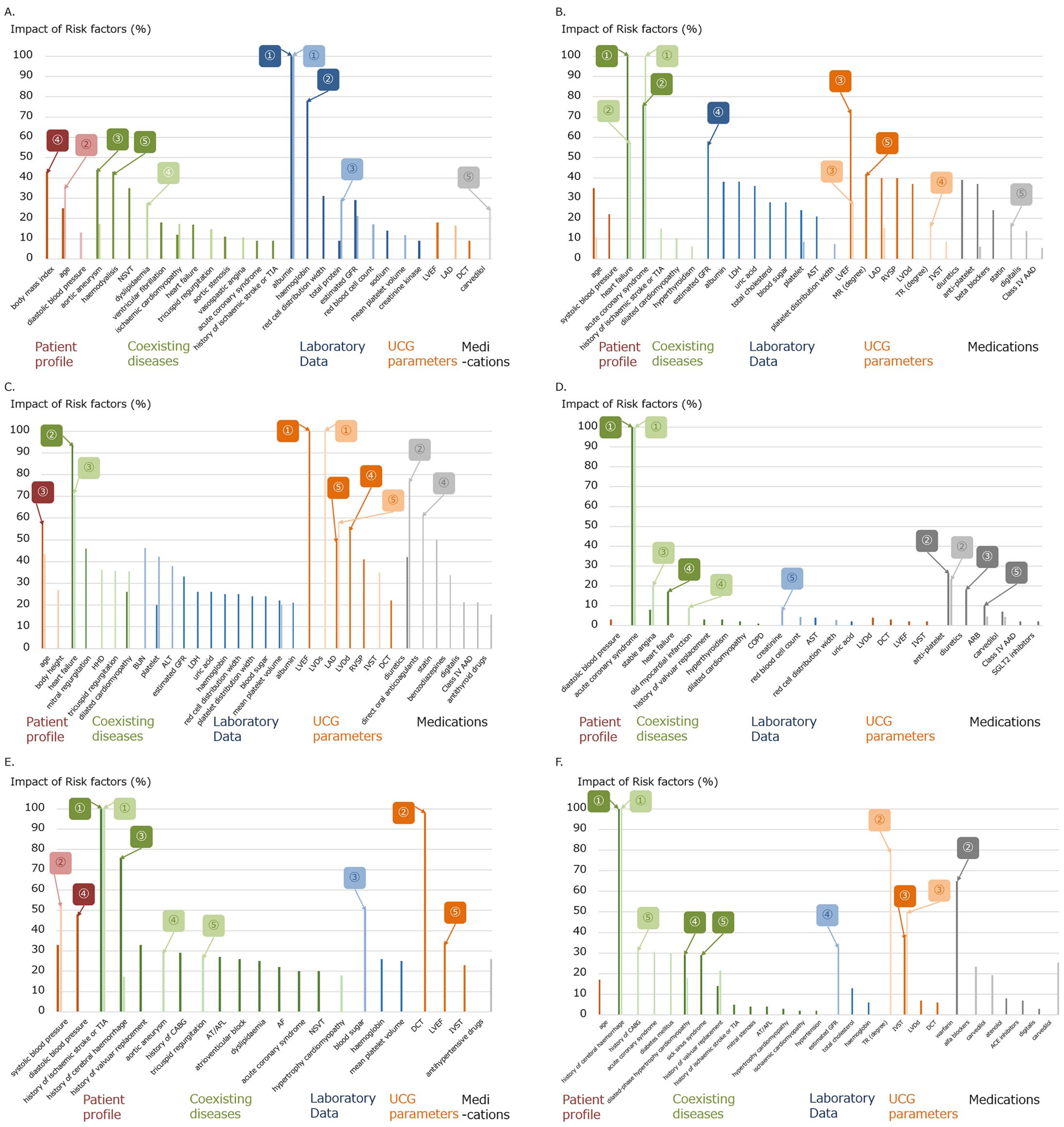

Fig 1. Impacts of risk factors selected in the prediction models with machine learning (ML) and logistic regression (LR) for six patient outcomes. (A) All-cause mortality: The areas under the curve (AUC) for all-cause mortality by prediction models with ML and LR were 0.900 and 0.881, respectively, and the risk factor with the strongest impact was albumin (impact 100, reference) for both models, followed by hemoglobin (78) and age (36) in ML and LR models, respectively. (B) Cardiovascular events: AUC 0.848 and 0.831, respectively, the risk factor with the strongest impact was heart failure and acute coronary syndrome (ACS) (both, 100, reference), followed by ACS (76) and heart failure (57), respectively. (C) Heart failure events: AUC 0.912 and 0.907, respectively, the risk factor with the strongest impact was left ventricular ejection fraction and left ventricular dimension at end-systole (both, 100, reference), respectively, followed by heart failure (93) and diuretics (77), respectively. (D) ACS: AUC 0.879 and 0.884, respectively, the risk factor with the strongest impact was ACS (100, reference) for both models, followed by anti-platelet for both models (26 and 23 in ML and LR, respectively). (E) Ischemic stroke events: AUC 0.758 and 0.757, respectively, the risk factor with the

strongest impact was a history of ischemic stroke or transient ischemic attack (100, reference) for both models, followed by deceleration time (98) and systolic blood pressure (52), respectively. (F) Intracranial hemorrhage: AUC 0.753 and 0.726, respectively, the risk factor with the strongest impact was a history of intracranial hemorrhage (100, reference) for both models, followed by warfarin (65) and tricuspid regurgitation (79) in models with ML and LR, respectively.

https://doi.org/10.1371/journal.pone.0221911.g001

with the greatest impact in each model (top parameter) was mostly similar between ML and LR, but other risk factors were not necessarily consistent between them.

Modelling in ML

We used DataRobot in the present analysis, which automatically selected the model with the largest AUC among numerous ML models.

For ACS and intracranial hemorrhage events, the Elastic-Net classifier model was selected. This model was projected for data with strong multicollinearity. In predicting models for ACS and intracranial hemorrhage, the history of each event was determined as the top parameter,

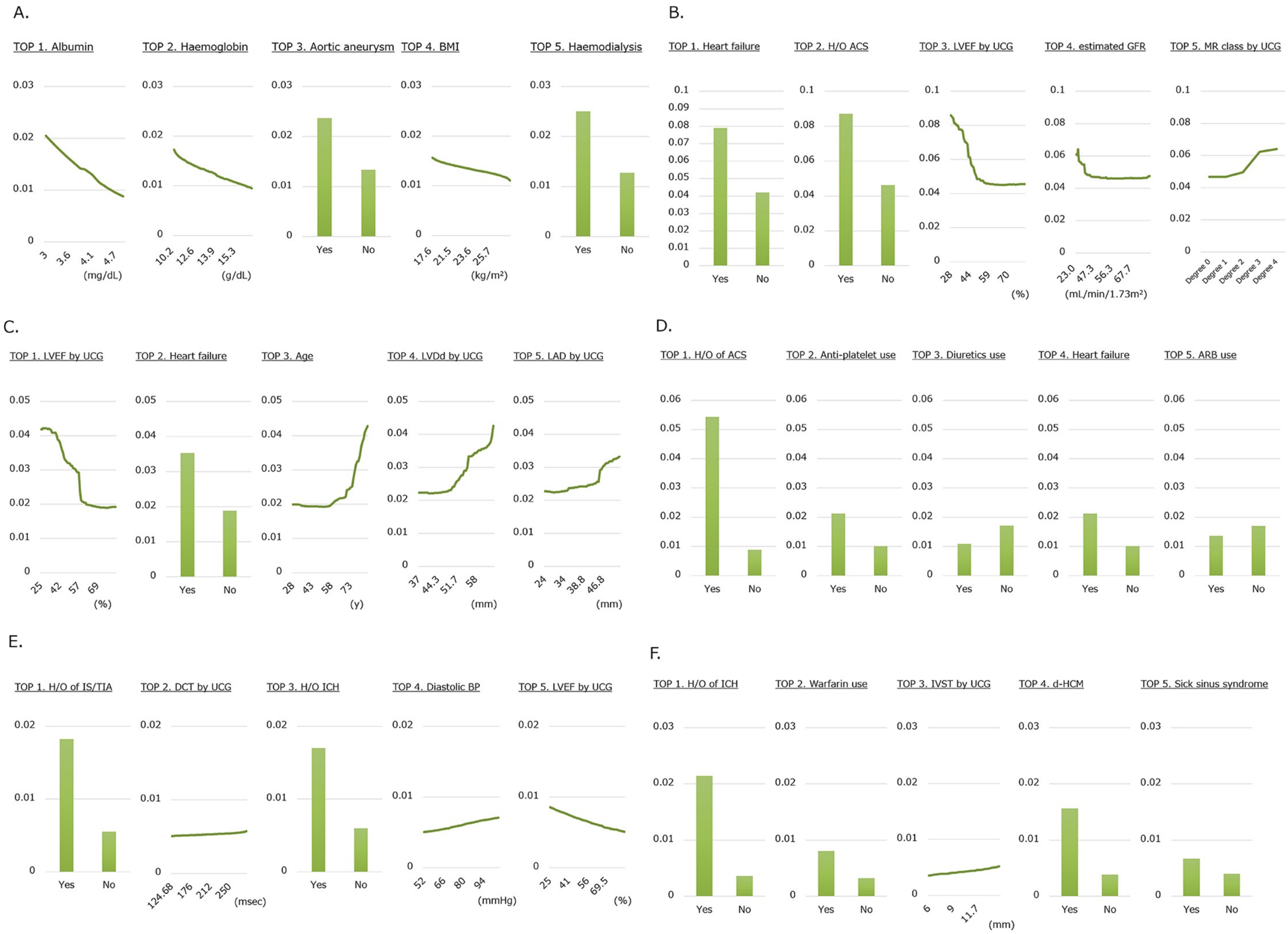

Fig 2. Relationships between parameters and incidence probability for six patient outcomes. The associations between the top five parameters by machine learning models and incidence probability of six patient outcomes are shown. The incidence probability was determined as partial dependence in each model. (A) All-cause mortality, (B) Cardiovascular events, (C) Heart failure events, (D) Acute coronary syndrome events, (E) Ischemic stroke events, and (F) Intracranial hemorrhage events.

https://doi.org/10.1371/journal.pone.0221911.g002

and the weighting of risk in the model was concentrated on the top parameter. In contrast, the weighing of the risk in other parameters in the model was very low. This distribution of impact may have been because that parameters indicating the progression of pathological condition (i.e., calcification or plaque of coronary artery for ACS, cerebral artery aneurism or micro-bleeds for intracranial hemorrhage) were not included in the present analysis, and therefore the history of each event played a role as a surrogate marker for which the weighing of risk was concentrated. As a consequent, the pattern of relationship among parameters became like to be a sparse representation, seen in the field of visual image reconstruction,[13, 14] for which Elastic-Net classifier model would fit well.[15]

The SVM model was selected for all-cause mortality and IS events. This model was projected to obtain a solution in a complex classification problem.[16] Especially, SVM is suitable for situations where appropriate and representative examples of all the different categories (classes) are available,[17] and as an advantage, SVM do not require linear relationships or independence between the parameters and thus are more suitable for clinical data classification.[18] As shown in Fig 2A and 2E (permutation importance for mortality and IS, respectively), multiple factors had strong impact on outcomes, which may be a reason that SVM suited better than Elastic Net classifier. On the other hand, the changes of the risks according to the change of each parameter were gradual (not having a threshold), which may be a reason that SVM suited better than random forest, which would suit better for parameters having a definite threshold.

For cardiovascular events and HF events, the random forest model was selected. This model was projected to obtain a solution with many parameters of similar effects and was widely used among non-linear model.[11] Similar to SVM, random forest do not require linear relationships or independence between the parameters and thus are suitable for clinical data classification. However, compared to SVM, random forest is more suitable for categorical data or consecutive values with a definite threshold, because its basic methodology is making categories with multiple layers. As shown in Fig 2B and 2C (permutation importance for cardiovascular events and heart failure events, respectively), multiple factors had strong impact on outcomes, and of note, the consecutive parameters had a definite threshold, which may be a reason that random forest suited better than SVM for these outcomes.

There are several possible explanations why the predictive capabilities represented by the AUCs were similar between ML and LR models for prognostic outcomes. First, the sample size was adequate for both ML and LR models, although ML can be applied for larger populations with greater numbers of predictors. Second, the numbers of risk parameters may be adequate for both of ML and LR models, and many of the consecutive parameters were mostly linearly correlated, which may make it difficult to distinguish the differences between ML and LR models. Third, given the relatively low event rate, the low signal-to-noise ratio may be another reason for the difficulty in distinguishing between the ML and LR models.[19]

Theoretically, it is expected that the superiority of ML in developing predicting models for prognostic outcomes will be more obvious in larger populations, especially with greater numbers of parameters and complex confounding factors or interactions. From another viewpoint, the problem of missing values may also be important. In the present study, albumin was commonly determined as the top predictor in both of ML and LR models. Although several recent studies have identified hypoalbuminemia as an independent predictor of mortality in CVD [20] (i.e., HF,[21–23] myocardial infarction,[24–26] or hemodialysis[27–29]), albumin was not included in most of the risk scores for mortality in CVD.[30–35] The insufficient recognition of albumin as a risk factor for mortality may be due to the strong interaction with other risk factors (i.e., hemoglobin and body weight) or missing values in previous observational studies due to the low attention to its risk. The ML method involves consideration of

interactions and the method of complementing missing values. Therefore, analysis by ML may lead to the discovery of new things by studying the past, where we can recognize the true risk in a data-driven manner.

Limitation

This study had several limitations. First, because our patients were from a single-centre cohort from a cardiovascular institute, the results should be interpreted carefully, and cannot be easily extrapolated to other populations. Second, as mentioned above, although the sample size seemed to be adequate for both ML and LR models, larger cohorts would be necessary to distinguish between the predictive capabilities of ML and LR models.

Conclusion

We reported our experience in the development of predictive models for cardiovascular prognosis by ML. ML could identify risk predictive models with good predictive capability and good discrimination of the risk impact.

Acknowledgments

We thank Shiro Ueda and Nobuko Ueda at Medical Edge Company, Ltd., for assembling the database by the Clinical Study Supporting System and Yukari Hashiguchi, Hiroaki Arai, and Hirokazu Aoki for data management and system administration. We also thank project members at Sigmaxyz, Inc., including Masashi Degawa, Suguru Ikezawa, and Norihito Iiyama, for project management and valuable insights for data analysis.

Dr. Suzuki received research funding from Mitsubishi Tanabe Pharm and Daiichi Sankyo. Dr. Yamashita has received research funding and/or lecture fees from Daiichi Sankyo, Bayer Yakuhin, Bristol–Myers Squibb, Pfizer, Nippon Boehringer Ingelheim, Eisai, Mitsubishi Tanabe Pharm, Ono Pharmaceutical, and Toa Eiyo.

Author Contributions

Conceptualization: Shinya Suzuki.

Data curation: Takuto Arita, Naoharu Yagi, Takayuki Otsuka, Hiroaki Semba, Hiroto Kano, Shunsuke Matsuno, Yuko Kato, Tokuhisa Uejima, Yuji Oikawa, Minoru Matsuhama, Junji Yajima.

Formal analysis: Shinya Suzuki, Tsuyoshi Sakama.

Investigation: Shinya Suzuki.

Methodology: Shinya Suzuki, Takeshi Yamashita, Tsuyoshi Sakama.

Resources: Takuto Arita, Naoharu Yagi, Takayuki Otsuka, Hiroaki Semba, Hiroto Kano, Shunsuke Matsuno, Yuko Kato, Tokuhisa Uejima, Yuji Oikawa, Minoru Matsuhama, Junji Yajima.

Software: Shinya Suzuki.

Supervision: Takeshi Yamashita.

Validation: Tsuyoshi Sakama.

Visualization: Tsuyoshi Sakama.

Writing – original draft: Shinya Suzuki.

Writing – review & editing: Takeshi Yamashita, Tsuyoshi Sakama.

References

1. Chen JH, Asch SM. Machine Learning and Prediction in Medicine—Beyond the Peak of Inflated Expectations. N Engl J Med. 2017; 376:2507–2509. https://doi.org/10.1056/NEJMp1702071 PMID: 28657867
2. Breslow NE. Analysis of Survival Data under the Proportional Hazards Model. International Statistical Review / Revue Internationale de Statistique. 1975; 43:45–57.
3. Deo RC. Machine Learning in Medicine. Circulation. 2015; 132:1920–1930. https://doi.org/10.1161/CIRCULATIONAHA.115.001593 PMID: 26572668
4. Weng SF, Reps J, Kai J, Garibaldi JM, Qureshi N. Can machine-learning improve cardiovascular risk prediction using routine clinical data? PLoS One. 2017; 12:e0174944. https://doi.org/10.1371/journal.pone.0174944 PMID: 28376093
5. Suzuki S, Yamashita T, Ohtsuka T, Sagara K, Uejima T, Oikawa Y, et al. Prevalence and prognosis of patients with atrial fibrillation in Japan: a prospective cohort of Shinken Database 2004. Circ J. 2008; 72:914–920. https://doi.org/10.1253/circj.72.914 PMID: 18503216
6. Suzuki S, Yamashita T, Otsuka T, Sagara K, Uejima T, Oikawa Y, et al. Recent mortality of Japanese patients with atrial fibrillation in an urban city of Tokyo. J Cardiol. 2011; 58:116–123. https://doi.org/10.1016/j.jjcc.2011.06.006 PMID: 21820280
7. Matsuo S, Imai E, Horio M, Yasuda Y, Tomita K, Nitta K, et al. Revised equations for estimated GFR from serum creatinine in Japan. Am J Kidney Dis. 2009; 53:982–992. https://doi.org/10.1053/j.ajkd.2008.12.034 PMID: 19339088
8. DataRobot [2019/3/14]. Available from: https://www.datarobot.com/.
9. Kang J, Schwartz R, Flickinger J, Beriwal S. Machine Learning Approaches for Predicting Radiation Therapy Outcomes: A Clinician's Perspective. Int J Radiat Oncol Biol Phys. 2015; 93:1127–1135. https://doi.org/10.1016/j.ijrobp.2015.07.2286 PMID: 26581149
10. Brooks Carthon JM, Jarrin O, Sloane D, Kutney-Lee A. Variations in postoperative complications according to race, ethnicity, and sex in older adults. J Am Geriatr Soc. 2013; 61:1499–1507. https://doi.org/10.1111/jgs.12419 PMID: 24006851
11. Breiman L. Random Forests. Machine Learning. 2001; 45:5–32.
12. Friedman J. Greedy boosting approximation: a gradient boosting machine. Ann Stat. 2001; 29:1189–1232.
13. Wright J, Yang AY, Ganesh A, Sastry SS, Ma Y. Robust face recognition via sparse representation. IEEE Trans Pattern Anal Mach Intell. 2009; 31:210–227. https://doi.org/10.1109/TPAMI.2008.79 PMID: 19110489
14. Wang L, Tong L, Yan B, Lei Y, Wang L, Zeng Y, et al. Sparse models for visual image reconstruction from fMRI activity. Biomed Mater Eng. 2014; 24:2963–2969. https://doi.org/10.3233/BME-141116 PMID: 25227003
15. Zou H, Hastie T. Regularization and Variable Selection via the Elastic Net. Journal of the Royal Statistical Society Series B (Statistical Methodology). 2005; 67:301–320.
16. Vapnik VN. An overview of statistical learning theory. IEEE Trans Neural Netw. 1999; 10:988–999. https://doi.org/10.1109/72.788640 PMID: 18252602
17. Unnikrishnan P, Kumar DK, Poosapadi Arjunan S, Kumar H, Mitchell P, Kawasaki R. Development of Health Parameter Model for Risk Prediction of CVD Using SVM. Comput Math Methods Med. 2016; 2016:3016245. https://doi.org/10.1155/2016/3016245 PMID: 27594895
18. Yu W, Liu T, Valdez R, Gwinn M, Khoury MJ. Application of support vector machine modeling for prediction of common diseases: the case of diabetes and pre-diabetes. BMC Med Inform Decis Mak. 2010; 10:16. https://doi.org/10.1186/1472-6947-10-16 PMID: 20307319
19. Ennis M, Hinton G, Naylor D, Revow M, Tibshirani R. A comparison of statistical learning methods on the Gusto database. Stat Med. 1998; 17:2501–2508. PMID: 9819841
20. Jung HY, Kim SH, Jang HM, Lee S, Kim YS, Kang SW, et al. Individualized prediction of mortality using multiple inflammatory markers in patients on dialysis. PLoS One. 2018; 13:e0193511. https://doi.org/10.1371/journal.pone.0193511 PMID: 29494637
21. Gotsman I, Shauer A, Zwas DR, Tahiroglu I, Lotan C, Keren A. Low serum albumin: A significant predictor of reduced survival in patients with chronic heart failure. Clin Cardiol. 2019; 42:365–372. https://doi.org/10.1002/clc.23153 PMID: 30637771
22. Nakano H, Omote K, Nagai T, Nakai M, Nishimura K, Honda Y, et al. Comparison of Mortality Prediction Models on Long-Term Mortality in Hospitalized Patients With Acute Heart Failure- The Importance of

Accounting for Nutritional Status. Circ J. 2019; 83:614–621. https://doi.org/10.1253/circj.CJ-18-1243 PMID: 30700666

23. Nishi I, Seo Y, Hamada-Harimura Y, Yamamoto M, Ishizu T, Sugano A, et al. Geriatric nutritional risk index predicts all-cause deaths in heart failure with preserved ejection fraction. ESC Heart Fail. 2019; https://doi.org/10.1002/ehf2.12405 PMID: 30706996
24. Xia M, Zhang C, Gu J, Chen J, Wang LC, Lu Y, et al. Impact of serum albumin levels on long-term all-cause, cardiovascular, and cardiac mortality in patients with first-onset acute myocardial infarction. Clin Chim Acta. 2018; 477:89–93. https://doi.org/10.1016/j.cca.2017.12.014 PMID: 29241048
25. Yang LJ, Feng YX, Li T, Jiao YR, Yao HC, Zhang DY. Serum albumin levels might be an adverse predictor of long term mortality in patients with acute myocardial infarction. Int J Cardiol. 2016; 223:647–648. https://doi.org/10.1016/j.ijcard.2016.08.251 PMID: 27567231
26. Plakht Y, Gilutz H, Shiyovich A. Decreased admission serum albumin level is an independent predictor of long-term mortality in hospital survivors of acute myocardial infarction. Soroka Acute Myocardial Infarction II (SAMI-II) project. Int J Cardiol. 2016; 219:20–24. https://doi.org/10.1016/j.ijcard.2016.05.067 PMID: 27257851
27. Ma L, Zhao S. Risk factors for mortality in patients undergoing hemodialysis: A systematic review and meta-analysis. Int J Cardiol. 2017; 238:151–158. https://doi.org/10.1016/j.ijcard.2017.02.095 PMID: 28341375
28. Chen CW, Drechsler C, Suntharalingam P, Karumanchi SA, Wanner C, Berg AH. High Glycated Albumin and Mortality in Persons with Diabetes Mellitus on Hemodialysis. Clin Chem. 2017; 63:477–485. https://doi.org/10.1373/clinchem.2016.258319 PMID: 27737895
29. Eriguchi R, Obi Y, Streja E, Tortorici AR, Rhee CM, Soohoo M, et al. Longitudinal Associations among Renal Urea Clearance-Corrected Normalized Protein Catabolic Rate, Serum Albumin, and Mortality in Patients on Hemodialysis. Clin J Am Soc Nephrol. 2017; 12:1109–1117. https://doi.org/10.2215/CJN.13141216 PMID: 28490436
30. Kannel WB, McGee D, Gordon T. A general cardiovascular risk profile: the Framingham Study. Am J Cardiol. 1976; 38:46–51. https://doi.org/10.1016/0002-9149(76)90061-8 PMID: 132862
31. Anderson KM, Wilson PW, Odell PM, Kannel WB. An updated coronary risk profile. A statement for health professionals. Circulation. 1991; 83:356–362. https://doi.org/10.1161/01.cir.83.1.356 PMID: 1984895
32. Zimmerman JE, Kramer AA, McNair DS, Malila FM. Acute Physiology and Chronic Health Evaluation (APACHE) IV: hospital mortality assessment for today's critically ill patients. Crit Care Med. 2006; 34:1297–1310. https://doi.org/10.1097/01.CCM.0000215112.84523.F0 PMID: 16540951
33. Miro O, Rossello X, Gil V, Martin-Sanchez FJ, Llorens P, Herrero-Puente P, et al. Predicting 30-Day Mortality for Patients With Acute Heart Failure in the Emergency Department: A Cohort Study. Ann Intern Med. 2017; 167:698–705. https://doi.org/10.7326/M16-2726 PMID: 28973663
34. Win S, Hussain I, Hebl VB, Dunlay SM, Redfield MM. Inpatient Mortality Risk Scores and Postdischarge Events in Hospitalized Heart Failure Patients: A Community-Based Study. Circ Heart Fail. 2017;10.
35. Chichareon P, Modolo R, van Klaveren D, Takahashi K, Kogame N, Chang CC, et al. Predictive ability of ACEF and ACEF II score in patients undergoing percutaneous coronary intervention in the GLOBAL LEADERS study. Int J Cardiol. 2019; https://doi.org/10.1016/j.ijcard.2019.02.043 PMID: 30846254
